# Right displacement of trachea to reduce right bronchial misplacement of left double lumen tube: a prospective, double-blind, randomized study

**DOI:** 10.1186/s12871-022-01850-y

**Published:** 2022-10-06

**Authors:** Jianqiang Guan, Wenxiu Zhu, Xue Xiao, Ziyan Huang, Jibin Xing, Ziqing Hei, Yihan Zhang, Weifeng Yao

**Affiliations:** Department of Anesthesiology, The Third Affiliated Hospital of Sun Yat-Sen University, Guangzhou, 510630 China

**Keywords:** Double-lumen endobronchial tube, One-Lung ventilation, Thoracic surgery

## Abstract

**Background:**

Misplacement of double-lumen endobronchial tubes (DLTs) during bronchial intubation, especially when bronchoscopy guidance is not applicable, threatens effective lung isolation and brings about airway injury during reposition. We aimed to examine whether a novel maneuver called right tracheal displacement (RTD) can reduce left-sided DLT misplacement during first-attempt intubation without bronchoscopy guidance.

**Methods:**

Patients that underwent thoracic surgeries requiring one-lung ventilation during November 2020 to January 2021 were recruited and randomized into control and RTD group, with 54 cases in each group. The primary outcomes included the incidence of DLT misplacement and the time to complete desired bronchial intubation. The secondary outcomes included mucosal injury, sore throat and hoarseness upon emergence and at 24 h post-operatively.

**Result:**

The incidence of DLT misplacement in RTD group was significantly lower compared to control group (0% vs. 16.7%) The time to complete bronchial intubation was also significantly shortened in RTD group compared to control (52.88 ± 9.36 s vs. 63.04 ± 20.02 s). The incidence of mucosal injury, sore throat and hoarseness were comparable between two groups.

**Conclusion:**

RTD maneuver can effectively improve the success rate of first-attempt proper DLT positioning and shorten the time required by bronchial intubation.

**Trial registration:**

This prospective, double-blind, randomized study has completed the registration of the Chinese Clinical Trial Center at 2/11/2020 with the registration number ChiCTR2000040212. It was conducted from 26/11/2020 to 31/7/2021 in third affiliated hospital of Sun Yat-sen university.

**Supplementary Information:**

The online version contains supplementary material available at 10.1186/s12871-022-01850-y.

## Background

Double lumen tubes (DLTs) designed by Robertshaw are standard instruments for one lung ventilation (OLV) that are applied the most [1–4] compared to bronchial blockers and other lung separating techniques. Due to the relatively complicated structure and larger diameter, endobronchial intubation with DLTs remains to be a clinical challenge. Some even consider difficult airway to be relative contraindication of DLTs intubation [5].

Videolaryngoscope can improve visualization of glottis and has been widely accepted clinically to increase the success rate of first-attempt intubation, both for ordinary single lumen tubes and double lumen tubes. However, it has been reported that videolaryngoscope tend to increase the resistance during DLTs intubation and also tend to increase the rate of misplacement [6–9]. A sequential rotating maneuver proposed by Sergio Bustamante et al. can facilitate DLTs intubation using GlideScope [10], but repeated rotation during intubation might increase the incidence of misplacement of left-sided DLTs.

Left-sided DLTs are generally preferred during OLV since there are additional requirements to avoid obstructing the orifice of the right upper lobe when using right-sided DLTs. However, the right main bronchus (RMB) is wider, shorter, and more vertical compared to the left main bronchus (LMB), resulting in a higher tendency for left-sided DLTs to be misplaced in the RMB. Repositioning of misplaced DLTs turns out to be challenging and threatens the patient with prolonged intubation time, hypoxia and higher risk of airway injury [11, 12]. Once misplacement of DLTs occurs, it would be challenging to adjust the DLTs to the desired location without additional technique or maneuver [13].

Whether the shape of DLTs can match the tracheal anatomy is a decisive factor of successful DLTs intubation. Anatomical parameters including tracheal diameter, diameter of RMB or LMB, respective trachea-bronchial angle and distance from carina to lobar bronchus have been studied as potential predictor of difficult DLTs intubation [12, 14]. It has also been reported that the tip of DLTs should be precurved to match the left main bronchus for successful bronchial intubation. Turning the head to the right (Head-turn maneuver) and counterclockwise rotation of the tube were the two techniques reported that can facilitate blind repositioning of misplaced left-sided DLTs [12, 13, 15, 16]. The Head-turn maneuver shifts the larynx to the same direction in relation to the carina, thereby aligning the axis of LMB with that of trachea and providing a straighter passage into LMB for DLT. Nevertheless, the alignment of larynx-trachea-main bronchus can also be influenced by anatomy of cervical spine and tissues enclosed around the neck. The head-turn maneuver is not suitable in patients co-morbid with cervical pathology that should minimize rotation of the neck. On the other hand, the cricothyroid joint is the articulation between the thyroid and cricoid cartilages, which can be easily palpated and relatively mobilized to facilitate tracheal displacement without additional movement of the neck. We postulate that pushing the cricothyroid joint to the right, a right tracheal displacement (RTD) maneuver, can shift and improve the laryngo-tracheo-LMB alignment and reduce misplacement of left-sided DLTs during intubation.

Fiberoptic bronchoscopic (FOB) or video flexible intubation scope (VFIS) guidance is a default technique for examining and adjusting the placement of DLTs in developed regions including Europe and the United States. However, such devices many not always be available due to sterilization requirement and high cost of maintenance, especially in less developed regions. In patients with unconventional airway anatomy, repositioning of misplaced DLTs can be difficult despite the help of VFIS guidance. Novel maneuver or technique that is not limited by anatomy heterogeneity, availability of VFIS or size of DLTs should be invented as a back-up plan for FOB guidance in these situations. In this study, we aimed to elucidate whether RTD maneuver can facilitate DLTs intubation and reduce DLTs misplacement, during which we quantified the force and distance required by RTD maneuver to illustrate the underlying mechanical and geometric mechanism.

## Methods

### Ethics

The study protocol was approved by the institutional review board of the Third Hospital of Sun Yat-sen University on 2th November 2020, reference number – [2020]02–189-01. The study protocol was registered in the Chinese Clinical Trial Register (ChiCTR2000040212; principal investigator: Weifeng Yao; date of registration: Nov 25, 2020; http://www.chictr.org.cn/). The report of this study adheres to the Consolidated Standards of Reporting Trials (CONSORT) statement. Written consent was obtained from the eligible participants a night before surgery. The study was undertaken in the Department of Anesthesiology, third Affiliated hospital of Sun Yat-sen University.

### Design

This is a randomized, controlled, double blind non-inferiority study. After obtaining written informed consents, the patients with an ASA physical status I or II, age between 20 to 75 years old, a height within 155 to 178 cm who were schedule for elective thoracic surgery requiring the placement of a left-sided DLT during November 26^th^ 2020 to July 31^st^ 2021, were enrolled. We excluded patients suspected of difficulties with airway management such as Mallampati score of 4 or small mouth opening (less than 3 cm), any bronchial malformation, obstruction or injury manifested in the medical records. Other exclusion criteria included patients with learning difficulties or limited understanding of the local language, length of one’s surgery expected to exceed 6 h, patients with a history of gastroesophageal reflux, and patients scheduled for tracheostomy or prolonged postoperative ventilation support in the intensive care unit (ICU). In addition, patients with preexisting hoarseness, sore throat, or morbidity with cervical spine were also excluded in the current study.

Using an internet-based computer program (https://www.randomizer.org), patients were randomly assigned to one of the two groups: Group of maneuver (right trachea displacement) or sham maneuver.

### Anesthetic management

The DLT size was selected according to the sex and the height of patients [17, 18]. We used a 37 Fr DLT for men around 160 to 178 cm tall and for women taller than 165 cm; a 35 Fr DLT for men shorter than 160 cm and for women around 153 to 165 cm tall. The tube was lubricated well, and the stylet of the tube was curved so that the distal part of the DLT, around 10 to 12 cm, The DLT was precurved to an angle around 120 to 135 degree between the main tube and the tip [19].

On arrival in the operating room, standard monitoring was established, including arterial pressure, ECG, and pulse oximetry. General anesthesia was induced with 1–2 mg of midazolam, 3–5 ug/kg of sufentanil, 1.0–2.0 mg/kg of propofol and 1.5 mg/kg of cisatracurium, and maintained with continuous inhalation of 1.5–2.5% sevoflurane and infusion of 2–4 mg/kg/h propofol 2–4 mg/kg/h, cisatracurium 5–8 mg/h.

**Fig. 1:**
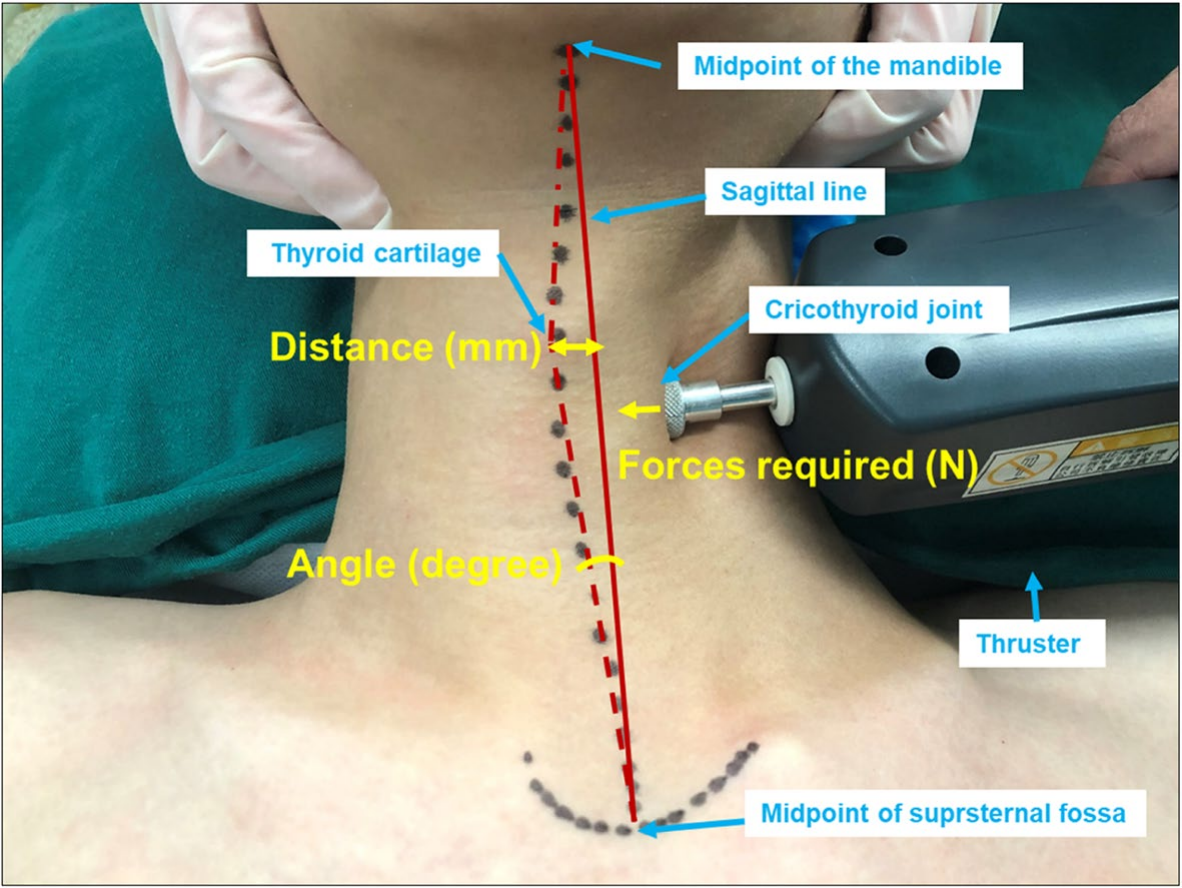
The image was obtained and labeled by our team to illustrate the measurement details of RTD maneuver. An example demonstration of how the potential displacement angle, distance and required force of the right trachea displacement were measured. The displacement distance of thyroid cartilage was the distance between the Adam’s apple and the sagittal midline. The displacement angle was the angulation between sagittal midline and the tie line between suprasternal fossa and the Adam’s apple. The force required by RTD maneuver was measured by a dynameter

The whole intubation procedure was jointly performed by 2 researchers. After successful induction, a senior anesthesiologist (Researcher A) pushed the cricothyroid joint to the right from the left side with his left thumb until a slight resistance was felt, and measured the displacement distance of the joint (the distance between the Adam’s apple and the sagittal midline) and the displacement angle (the angulation between sagittal midline and the tie line between suprasternal fossa and the Adam’s apple) (Deli Group Co. LTD, Shanghai, China). Then, a dynamiter was used to measure the force required to push the joint to the same displacement distance as previously measured (WD Electronics Co., LTD, Zhejiang, China) (Fig. 1).

After measuring the potential angulation of trachea that could have been achieved in each participant, a junior resident with less than 3 years of practice experience (Researcher B) perform the bronchial intubation with a left-sided DLT (Covidien Medtronic Brocho Cath Endobronchial Tube, Ireland) using a videolaryngo scope (UE, Zhejiang, China). Sequential rotation or a ‘left-to-right’ rotation maneuver was performed [10]. When the tip of the double-lumen tube had passed through the vocal cords, the stylet was removed, the DLT was rotated 180°counterclockwise to direct the tracheal lumen anteriorly, and then the tube was advanced until the tip of the tracheal lumen passed just beyond the vocal cords. Afterwards, the DLT was re-rotated 90 degree clockwise to align the bronchial lumen with the LMB and researcher B suspended the procedure and informed researcher A.

In the RTD group, researcher A at this time pushed the cricothyroid joint to the right transversely to an extent as has been described previously, whose action was covered by a blanket over the patient’s neck. Then researcher A asked researcher B to deliver the DLT until resistance was felt. In the sham maneuver group (control group), researcher A merely palpated the joint and pretended to push, then asked researcher B to go on with the procedure.

Once resistance was felt and the DLT was assumed to be in the bronchus, a video flexible intubation scope (VFIS, Denmark, AMBU aScope3) was immediately used to examine the placement of DLT and to adjust the depth of DLT so that the bronchial balloon was just below carina. If misplacement of the left-sided DLT in the right main bronchus was identified, the tube was adjusted to the desired location under direct guidance of VFIS by researcher B. After confirmation of the correct DLT placement, researcher B continued to assess the mucosal injury of bronchus, trachea and larynx under VFIS, mainly to check if there was mucosal swelling or erythema, hematoma or bleeding. Then both the bronchial and tracheal cuffs were inflated and the tube was fixed with adhesive tapes and connected to a ventilator. A second confirmation of DLT location was performed under VFIS after the patient was adjusted to lateral position.

Prior to closure of the thorax, intercostal nerve block was done by local administration of 20 mL of 0.375% ropivocaine. The patients were then transferred to PACU for emergence and extubated once they regained spontaneous breathing and consciousness. The patients were then continued to be on monitor for 30 min before being transferred back to the ward.

### Study endpoints

The primary outcome was the incidence of DLT misplacement and total time required for intubation. DLT misplacement was diagnosed when the left-sided DLT ended up in the RMB. The time required for intubation was the interval from the contact of the laryngoscope with the teeth to the confirmation of correct DLT placement. The secondary outcome included postoperative incidence of hoarseness and sore throat. Hoarseness was defined as an acoustic quality that was different from the previous voice quality of the patient [20] and sore throat as continuous throat pain [21]. Other parameters including Mallampati score, Cormack-Lehane classification of glottis exposure during laryngoscopy [22]. The parameters of tracheal displacement were collected as previously stated. The mucosal injury examined under VFIS included the following aspects: normal, mild swelling or erythema, hematoma, bleeding [23].

Before the patient left PACU and 24 h after the surgery (POH24), a third researcher (Researcher C), who was blinded to the randomization, inspected the patients for hoarseness and sore throat. Hoarseness was graded by 5 levels as follows [24]: none, which means no hoarseness; mild, which means it could be felt by the patient; moderate, which means it was obvious to the patient; severe; aphonia. Sore throat was graded by 4 levels as follows [25]: none, no sore throat; mild, pain with deglutition; moderate, pain present constantly and increasing with deglutition; severe, pain interfering with eating and requiring analgesic medication.

### Statistical methods

A pilot study was conducted to determine the sample size. We performed either RTD maneuver or conventional technique on 40 patients who were evenly distributed into 2 groups. The success rate of first-attempt blind DLT intubation in the pilot study was 85% (17/20), and the rate of RTD group was 95% (19/20). The sample size was calculated by Power Analysis & Sample Size (*PASS, 15.0*) as 98. Considering a 10% dropout, a group size of 54 was needed to detect a difference with a power of 0.8 and an α-level of *0.05*.

Data were analyzed using SPSS 20.0. The data collected in this research were all independent data. We assessed whether these data follow normal or non-normal distribution by the Kolmogorov–Smirnov test. Data that follow normal distribution were represented by mean ± (SD). Data of non-normal distribution were represented by median ± interquartile range. Categorical variables were represented by frequency and percentage. The two-sided Student t-test (normal distribution) or Mann–Whitney U-test (non-normal distribution) was used for inter-group comparison. Frequencies were analyzed with the Chi-Squared test or Fisher’s exact test as appropriate. All statistical analyses were performed using SPSS 20.0 software (SPSS, Chicago, IL, USA); a *p* value of < 0.05 was considered statistically significant.

## Results

### Baseline data, participant flow and recruitment

**Fig. 2:**
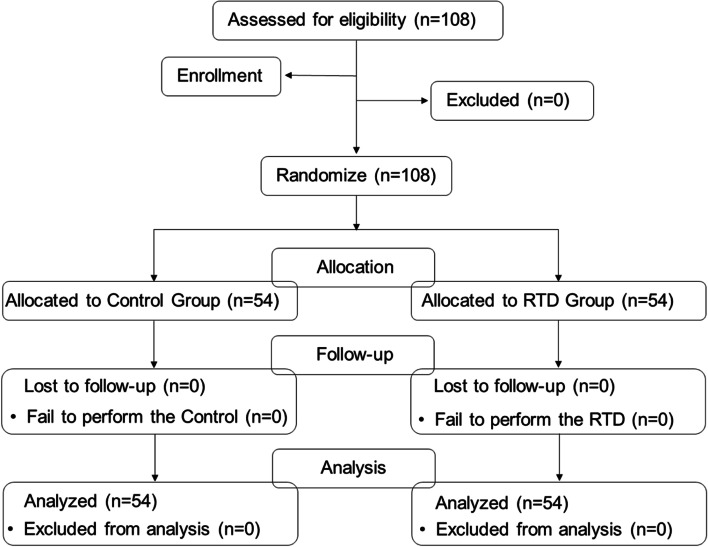
A flow chart of the current trial

Our participants mainly consist of a group of 54.6% male, with mean age of 50.65 years old. ASA grade, preoperative lab results, mouth opening, thyromental distance and cricoids-sternal distance were comparable between RTD group and control (Table 1). The flexibility of trachea, examined by preoperative assessment of the angle and distance of tracheal displacement, and the force required for trachea displacement were comparable between the two groups. The participant flow is demonstrated in Fig. 2. We recruited a total of 108 participants, and distributed 54 in each group respectively. There were no participant losses or exclusions throughout the trial. All the participants recruited were enrolled and completed the follow-up. The trial was ended after we have enrolled the presumed samples size.

**Table 1:** characteristics and intraoperative variables

	Control	RTD	*P* value
Age (y)	49.85 ± 14.30	51.46 ± 13.56	*P *= 0.549
Sex (male, n)	30/54 (55.5%)	29/54 (53.7%)	*P* = 0.847
Height (cm)	164.59 ± 8.11	162.50 ± 7.44	*P* = 0.165
Weight (kg)	62.70 ± 12.61	59.62 ± 10.05	*P* = 0.164
Mallampati grade (1/2/3/4)	28/19/7/0	29/21/4/0	*P* = 0.722
Displacement distance (mm)	13.20 ± 1.52	13.31 ± 1.54	*P* = 0.730
Forces required (N)	5.70 ± 0.79	5.67 ± 0.87	*P* = 0.852
Displacement angle (degree)	11.91 ± 1.59	12.19 ± 1.87	*P* = 0.392
Cormack-Lehane classification (1/2/3/4)	33/17/4/0	38/14/2/0	*P* = 0.515
Size of DLT (Fr)	34/20	41/13	*P* = 0.210
Type of surgery (VATS/ thoracotomy)	53/1	54/0	*P* = 1.000
Surgical side (left/right/others)	16/33/5	15/32/7	*P* = 0.919
Duration of surgery (min)	115.07 ± 61.63	120.31 ± 64.86	*P* = 0.667
Duration of anesthesia (min)	211.26 ± 68.99	215.37 ± 76.32	*P* = 0.770
PCA	22/54(40.7%)	19/54(35.2%)	*P* = 0.692

Displacement distance: the distance between the Adam’s apple and the sagittal midline when pushing the cricothyroid joint; Displacement angle: the angulation between sagittal midline and the tie line between suprasternal fossa and the Adam’s apple when pushing the cricothyroid joint

*DLT* Double lumen tube, *VATS *Video-assisted thoracic surgery, *PCA* Patient controlled analgesia

### Primary outcomes

**Fig. 3:**
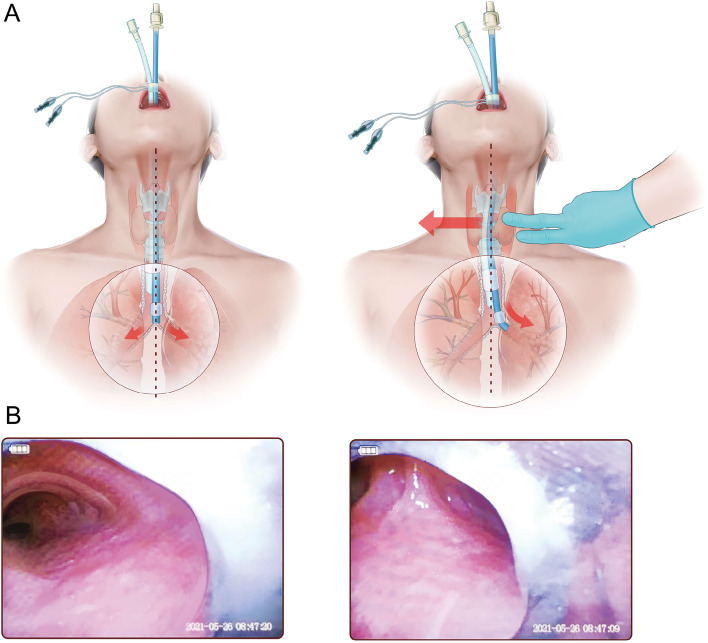
A diagram was draw by our team to illustrate the technical details of RTD maneuver. **A**: A graphical description of RTD maneuver. RTD maneuver improves the alignment of trachea and LMB, thus facilitating the passage of left-sided DLT into the desired location. **B**: Bronchoscopic finding of the changes of trachea-LMB alignment. The orifice of LMB moves towards the center of the view when applying RTD maneuver, which proves that the alignment of trachea and LMB is improved

The incidence of DLT misplacement in the control group was 16.67%, while we did not encounter any case of DLT misplacement in RTD group (16.67% *vs* 0%, *p* = *0.002*) (Table 2). The 9 cases of misplacement in the control group were all instantly corrected by one attempt. The time required for successful final placement of DLT was significantly longer in the control group (mean = 63.04 ± 20.02 *vs* 52.88 ± 9.36 s, *p* < *0.001*). Meanwhile, the orifice of LMB, which usually appeared in the left side of the view when centering carina under VFIS, would move towards the center of view after implementing RTD maneuver (Fig. 3 and supplemental vedio-01).

**Table 2:** Primary outcome

	Control	RTD	*P* value
Misplacement of DLT (n)	9/54	0/54	*P* = 0.002
Total time of intubation (s)	63.04 + 20.02	52.88 + 9.36	*P *< 0.001

*DLT* Double lumen tube

### Secondly outcomes

Minor mucosal injuries were observed in both groups including mucosal swelling, mild erythma and self-limited bleeder, with no significant difference among the two groups. Around a half of the participants presented mild sore throat in the PACU, most of which were alleviated on 24 h post-operatively (POH24). The incidence of post-operative hoarseness and sore throat were also not significantly different between the 2 groups. No adverse event or severe adverse event was identified throughout the study (Table 3).

**Table 3:** The incidence and the severity of mucosal and pharyngeal injury

	Control (*n* = *54*)	RTD (*n* = *54*)	*P* value
Mucosal injury (n)			
Non	26	29	*P* = 0.838
Erythema	8	5	
Hematoma	10	11	
Bleeding	10	9	
Sore throat upon emergence (n)			
Non	24	37	*P *= 0.028
Mild	28	15	
Moderate	2	2	
Hoarseness upon emergence (n)			
Non	48	45	*P* = 0.776
Mild	5	8	
Moderate	1	1	
Sore throat at POH24 (n)			
Non	50	51	*P* = 1.000
Mild	4	3	
Moderate	0	0	
Hoarseness at POH24 (n)			
Non	51	52	*P* = 0.618
Mild	3	1	
Moderate	0	1	

POH24: 24 h after the surgery

## Discussion

In our study we showed that right trachea displacement greatly enhanced the first-time attempt of blind bronchial DLT intubation. The direction of the tube from the glottis to the final destination, that is, the left main bronchus, determines whether the left-sided DLT can be delivered correctly with first-attempt blinded intubation. The reasons of right bronchial misplacement of left-sided DLT include larger diameter and straighter angle of RMB. Moreover, tracheal bifurcation, marked by identification of carina, usually locates to the left of the tracheal midline, which is a consequence of the asymmetrical structure of respiratory tract. All though the left-sided DLTs are specifically designed to curve the tip to an angle of 147 to 150 degree to conform to human anatomy [12], the rate of left-sided DLTs misplacement into the RMB using traditional laryngoscope ranges from 4.5 ~ 8.8% [11, 19], which can be aggravated to 10% when using videolaryngoscope [8, 11].

Headturn maneuver is a major technique to reduce the risk of misplacement of left-sided DLTs [12, 13, 15, 16]. This technique mainly involves raising and flexing the patient’s head and neck, turning the patients face to the right after the tracheal balloon is passed beyond the glotti [26], and delivering the tube until resistance was encountered. The “headturn” maneuver was probably inspired by the experience of endoscopist from bronchoscopy when examining left mainstem bronchus, which shift the larynx to the right in relation to carina. The head-turning maneuver requires that further delivery of DLTs can only be continued after the tracheal balloon was in the trachea and the turning is completed to minimize mucosal injury [13]. Although the headturn maneuver improve the tracheal-LMB alignment by shifting the position and direction of larynx, the alignment between larynx and trachea remains to be uncertain. However, the headturn maneuver can be limited in patients whose cervical range of motion is restraint or who require cervical spine stabilization. Cricothyroid joint, on the other hand, can be easily palpated and mobilized without much anatomical or pathological limitation. This technique can be done by a single operator, with one’s right hand delivering the DLT, and the left hand pushing the trachea to an extent when mild resistance was felt. The force was around 5.6 N when the cricothyroid joint was displaced for about 12 mm, forming an angle around 12 degrees between the larynx and the sagittal plane, which facilitates the alignment of trachea and the orifice of LMB, helping the direction of left-sided DLT towards the LMB. We also confirmed improved alignment of trachea-LMB by direct visualization via bronchoscopy, which demonstrated that the orifice of LMB would move towards the center of the view when applying RTD maneuver, thus reducing misplacement of the tube. Moreover, by palpating the trachea when delivering the tube, the practitioner can have a strengthened feeling of the resistance encountered with both hands during DLT insertion, and possibly be gentler in action and be better alarmed by abnormal resistance during intubation.

Correct selection and pre-curving of DLT facilitates correct placement. The bronchial tip angle of 32-Fr left sided DLT (153–155°) appears to be more obtuse than those of larger-sized DLTs (147–150°) [11]. Seo J. et al*.* reported that augmentation of the curved tip to 135° reduced the right bronchial misplacement and facilitated intubation without aggravating airway injury [19]. However, most anesthetists have already tried to bend this angle to a similar extent to facilitate the passage of DLT through vocal cord in the first place, and the curved angle can hardly maintain unchanged during bronchial intubation. We should also avoid advancing the tube before the rotation was completed, otherwise the route of DLT and the final direction of the tip may differ from original plan.

In the current study, the extent of mucosal injury was comparable between the two groups and was similar of that reported in the past studies. Most of these injuries was self-limiting and can be ameliorated in 24 h without additional intervention. Rotation of larger DLTs may cause more severe injury. Factors including sex, history of smoking, tracheal diameter, size of DLTs and lack of intubation experience are all reported risk factors of airway injury [27, 28]. The tip of DLTs scratching the mucosa is also a main cause of airway injury. We chose to examine mucosal injury right after intubation since these signs were more obvious at this time rather then examing them after extubation. We could identify the major injury through the semitransparent tube wall, but a potential limitation of this method was that some trivial injury might have been covered by the tube. A second check after the tube is removed can offer a more thorough view of the main bronchus, but performing extra bronchoscopy in PACU in extubated patients may require extra sedation, otherwise it would be very uncomfortable. However, such extra sedation may threaten the airway patency of these patients, so we did not design such procedures in the current study.

This trial holds several limitations as follows. First of all, we did not examine the RTD maneuver with DLT above the size of 39 Fr or below the size of 32 Fr. Although techniques such as RTD might be of help for blinded intubation of small-size DLTs to which fiberoptic scope may not always be applicable or available, the effectiveness of RTD maneuver with these sizes requires further investigation. Secondly, the force we applied during trachea displacement was around 5 N, reaching a displacement distance around 1.2 cm. This distance and force was determined by the practitioner’s feeling of mild resistance that encountered when pushing the trachea, which may be divergent among operators and patients. Higher forces would result in a further displacement distance but might increase the mucosal injury caused by friction from the tip of DLTs. Finally, although we have identified that the orifice of LMB, which usually appeared in the left side of the view when centering carina under bronchoscopy, would move towards the center of view after implementing RTD maneuver. Our postulation that RTD maneuver may promote the alignment of LMSB with the DLT path would require more decisive imaging evidence including X-rays or CT scans of the coronal plane. In this preliminary study we did not design such imaging procedure in order not to interfere with the surgery. We will consider designing more specific trials to verify this theory in the upcoming researches.

## Conclusion

The right trachea displacement can effectively reduce the right bronchial misplacement of left-sided DLTs without increasing airway injury, which could be a recommended maneuver during left-sided DLT intubation.

## Supplementary Information

**Additional file 1. **

## Data Availability

The datasets used and/or analyzed during the current study are available from the corresponding author on reasonable request.

## Abbreviations

DLTs: Double-lumen endobronchial tubes
RTD: Right tracheal displacement
OLV: One lung ventilation
RMB: Right main bronchus
LMB: Left main bronchus
VFIS: Video flexible intubation scope
POH24: 24 Hours after the surgery
PACU: Postanesthesia care unit
